# Cross-lagged analysis of the effect of adolescent loneliness on collective responsibility and self-improvement values

**DOI:** 10.3389/fpsyg.2026.1781731

**Published:** 2026-04-23

**Authors:** Lili Lan, Xiaofeng Wang

**Affiliations:** 1 Shanghai Youth College of Management, Party-Mass Work Division, Shanghai, China; 2 Student Mental Health Education Centre, Shanghai University of Political Science and Law, Shanghai, China

**Keywords:** Chinese adolescent, collective responsibility value, cross-lagged analysis, loneliness, self-improvement value

## Abstract

A cross-lagged study design was used to explore the influence of loneliness on adolescent values. The Chinese Adolescent Values Questionnaire and the Loneliness Scale were both administered twice, 1 year apart, to 403 7th and 11th graders living in Shanghai. The results showed that overall, loneliness at Time 1 (T1) significantly negatively predicted the adolescent values of collective responsibility (CR) and self-improvement (SI) at Time 2 (T2). Furthermore, differences were found in the adolescents’ relationship between loneliness and SI according to gender and age. Specifically, 7th graders’ SI at T1 negatively predicted their loneliness at T2, while 11th graders’ SI at T1 positively predicted their loneliness at T2. Additionally, boys’ loneliness at T1 significantly negatively predicted their SI at T2. These results suggest the possible role of loneliness in reducing adolescent identification with the values of CR and SI. Educators should pay attention to changes in adolescent values and provide them with education and guidance to prevent them experiencing chronic loneliness. Researchers should also be more sensitive to the development of values during adolescence, particularly those of specific ages or genders, such as in older males.

## 1 Introduction

Loneliness is a negative psychological state which stems from a difference between one’s existing level of social relationships and their desired level of such social relationships. Loneliness is often accompanied by negative emotions such as isolation, helplessness, and depression (Igami et al., 2023; Ren et al., 2020). Adolescents are in a crucial stage of self-awareness development. Their dependence on their parents is progressively diminishing, and they are eager to establish their own social connections. Many adolescents may begin to experience loneliness when there is a gap between their desired level of social interactions and their actual level of interactions (Zhang et al., 2019). One study investigated the experience of loneliness in adolescents across 37 countries between 2012 and 2018, and found that loneliness was increasing in adolescents in 36 of these countries (Twenge et al., 2021). Research on adolescent loneliness in China suggests that among Chinese adolescents, levels of loneliness have increased year by year over the past two decades (i.e., from 2001 to 2019; Peng et al., 2023).

Loneliness is an important influence on adolescent psychosocial adjustment (Yang et al., 2026; Hu et al., 2019). The results of one meta-analysis suggest that loneliness is a significant predictor of suicidal thoughts and behaviors in adolescents (McClelland et al., 2020). In individuals, loneliness is closely associated with a spiritual absence and a lack of a sense of meaning in life (Ma et al., 2023; Zhang et al., 2020), which are understood as being major reasons as to why it has so many negative effects on adolescent development. When adolescents experience chronic loneliness, they begin to lose their sense of purpose in life, feeling unsure of where they want to be or what they want to accomplish in the future, and begin to lose their sense of their life’s significance. It may also lead adolescents to experience confusion or absence in terms of their value identity. Values are certain states, objects, goals, or behaviors that are desirable, which exist beyond specific situations and can be used as criteria when making judgments or choices between a range of behavioral options.

### 1.1 Value identity and categorization of Chinese adolescents

Adolescence is a critical period in the development of values, during which their values become set for the remainder of their lives (Gubbels et al., 2024; Vecchione et al., 2016). For today’s Chinese adolescents, the classification of group-oriented and self-oriented values captures the core tension in their value identity development (Liu et al., 2018; Wang et al., 2018).

In this study, collective responsibility (CR) and self-improvement (SI) were chosen to represent group- and self-orientation values, respectively. CR emphasizes caring about the well-being of others and abiding by collective rules, reflecting traditional Confucian values of social harmony and group belonging. SI emphasizes individual goals, self-discipline, competition, and continuous self-transcendence, reflecting the increasing emphasis on individual achievement in modern Chinese society (Li X. R. et al., 2018; Li D. et al., 2018). These two values were selected because they represent the most salient dimensions of group- and self-orientation in the Chinese cultural context and have demonstrated significant associations with adolescent loneliness in prior cross-sectional research (Li X. R. et al., 2018; Li D. et al., 2018; Liu et al., 2018).

To understand how loneliness influences value identification, we draw upon self-determination theory (SDT; Ryan and Deci, 2022), which posits that human motivation and well-being depend on the satisfaction of three basic psychological needs: autonomy, competence, and relatedness. Loneliness represents a fundamental deficit in relatedness need satisfaction. According to SDT, when relatedness needs are chronically unmet, individuals may experience diminished motivation to engage with social groups and collective goals, thereby reducing their identification with group-oriented values such as collective responsibility. Similarly, chronic loneliness may undermine competence need satisfaction, as lonely adolescents often experience lower self-efficacy and self-esteem (Liu et al., 2018), which may in turn reduce their motivation for self-improvement and achievement. Furthermore, we integrate Bardi and Goodwin’s (2011) dual-route model of value change, which distinguishes between intentional and automatic pathways. We propose that loneliness influences values primarily through automatic pathways—specifically through priming and self-concept consistency maintenance—whereby the emotional and cognitive experience of loneliness primes individuals to distance themselves from values that require social connection or personal agency.

### 1.2 The relationship between adolescent loneliness and values

Loneliness is a key emotion experienced during adolescent development and has a profound impact on adolescent mental health and social adjustment (Hunter et al., 2024). However, few studies have focused on the influence of adolescent loneliness on values. Some longitudinal studies have shown that pro-social behavior positively predicted self-transcendent values (Vecchione et al., 2016), while aggressive behavior negatively predicted self-transcendent values 1 year later (Benish-Weisman, 2015). This suggests that the relationship between values and behavior is not only “top-down” but may also be “bottom-up.,” with adolescent behaviors also potentially having an impact on the formation and development of their values. However, behavior is always accompanied by emotions, such as pro-social behavior being associated with positive emotions such as empathy, or aggressive behavior being associated with loneliness (Chen F. et al., 2023; Chen Z. et al., 2023). Therefore, loneliness may also play an important factor in influencing adolescent values identity and development.

Many cross-sectional studies have shown a link between loneliness and adolescent values. For example, in one study, loneliness was seen to be negatively associated with self-transcendence and openness to change in values, yet loneliness was also positively associated with self-enhancement and conservation values (Liu et al., 2021). A relationship has also been identified between adolescent loneliness and family cultural values, with one study finding that parental group-orientation values positively predicted loneliness through adolescents’ CR; meanwhile, parental self-orientation values and maternal group-orientation values positively predicted loneliness through adolescents’ SI (Li et al., 2023).

One reason why loneliness may have a long-term impact on adolescents’ value identity might be because loneliness is an emotion, and emotions can influence the direction and intensity of one’s behavior by enhancing motivation. For example, positive emotions may enhance one’s motivation to seek rewards, while negative emotions may stimulate avoidance behaviors (Ryan and Deci, 2022). In addition, emotions can influence behavior by moderating the relationship between intrinsic and extrinsic motivation. The human value system is also based on the motivational system; for example, Schwartz’s value theory suggests that values are conscious responses driven by three motivations – the needs of the biological organism, requisites of coordinated social interaction, and the survival and welfare needs of groups – and the way in which they impact an individual’s socialization and cognitive development (Vecchione et al., 2016).

Individuals experiencing loneliness may be more likely to identify with values such as group isolation or social rejection, or feel distanced from emotions such as acceptance, compassion, and caring. Humans are by nature socially connected beings. Throughout their lives, we pursue a sense of meaningful connection, encompassing belonging, interpersonal harmony, and interpersonal satisfaction (Kaya et al., 2024; Lambert et al., 2013). Interpersonal rejection, neglect, and social isolation all contribute to the loss of one’s a sense of connectedness and belonging, and as one experiences an increased sense of loneliness, they will begin to experience a sense of group alienation (Li et al., 2025; Xiong et al., 2021). We therefore hypothesized that loneliness associates with a decrease in adolescent identification with CR, in that they would have a reduced level of concern for group well-being and show less willingness to take on group responsibility.

Similarly, the level to which individuals may identify with SI may also change depending on whether they are experiencing emotions such as boredom and depression, or of excitement and arousal. Previous research has found that loneliness is significantly associated with depression in individuals (Chen F. et al., 2023; Chen Z. et al., 2023; Zhang et al., 2019), and that loneliness predicts increased depression (Ren et al., 2020). Additionally, adolescents experiencing loneliness have been shown to be more likely to adopt negative coping styles, such as not actively seeking support, avoiding sleepovers with friends, and avoiding difficulties, in addition to experiencing increased depression and emptiness. Loneliness is also associated with lower self-esteem in adolescents, in that adolescents with high loneliness have been found to have lower senses of self-worth and self-efficacy (Liu et al., 2018). In consideration of these findings, we hypothesized that loneliness may lead to a decrease in adolescent identification with SI.

### 1.3 Gender and age differences in the relationship between loneliness and values in adolescents

Studies of gender differences in adolescent loneliness and their values have had inconsistent results (Zhang et al., 2019; Wang et al., 2018), making it difficult to formulate hypotheses regarding the effects of gender differences. Girls have typically reported more social loneliness, but this could be because they tend to be better at expressing their feelings through conversation and thus more likely to receive emotional support (Parlikar et al., 2023). Girls also have been seen to have significantly higher perceptions of unfulfilled levels of important relationships than boys. On the other hand, boys may be more inclined to hide feelings of loneliness (Parlikar et al., 2023), despite the fact that they may experience significant social anxiety and negative emotions when they are lonely. This difference may be related to gender role stereotypes, whereby boys are expected to appear stronger and more independent, leading to a higher likelihood of identifying with values such as power and excitement. With these considerations in mind, we hypothesized that boys experiencing loneliness are more likely to identify less with CR and more with SI than girls.

Age has also been shown to have a significant effect on adolescent loneliness and value identity. One study showed that the level of loneliness among Chinese adolescents is increasing year by year (as measured from 2001 to 2019) (Peng et al., 2023). As adolescents get older, the values they identify with change, such as increasingly identifying with the value of self-direction or decreasing in identification with values of tradition or security (Daniel et al., 2020). However, few studies have focused specifically on the effect of adolescent loneliness on values. Therefore, we tentatively hypothesized that loneliness in older adolescents (i.e., those in higher grades) will cause them to identify more with SI and less with CR.

### The present study

To summarize, the present study attempted to evaluate the reciprocal relationships between loneliness and values according to age and gender differences. While previous research has explored how values may influence loneliness, the primary focus of this study is on loneliness as an antecedent of value development. However, given that cross-lagged panel analysis allows for the examination of bidirectional effects, we also report and interpret the reverse pathways (values → loneliness) to provide a comprehensive understanding of the longitudinal dynamics. This approach enables us to situate our primary findings within the broader context of value-loneliness reciprocal influences.

To better explore the causal relationships, we adopted a cross-lagged analysis in our research design, measuring participants’ loneliness and values twice, first at Time 1 (T1) and then at Time 2 (T2) 1 year later. In this study, the cross-lagged analysis method was chosen over others (e.g., regression analysis) as it can analyze the interactions of multiple variables at different points in time, allowing the researcher to consider both autocorrelation and causality of variables across the different time points (Kearney, 2017). The use of the cross-lagged approach in previous research on adolescent values and social adjustment has proven to be effective in exploring the interactions of values with pro-social and aggressive behaviors (Vecchione et al., 2016; Benish-Weisman, 2015). However, these previous studies have not focused on adolescent values and their psychological adjustment. The current study aimed to provide useful additions to the literature in this area by exploring the antecedent of loneliness and its impact on adolescents’ deep motivational system-values development.

Our hypotheses were as follows:

*Hypothesis 1*: Loneliness at T1 negatively predicts group- and self-oriented values at T2.

*Hypothesis 2*: There are gender-related differences in the relationship between loneliness and values, with boys experiencing loneliness at T1 being more likely to identify less with CR and more with SI than girls at T2.

*Hypothesis 3*: There are age-related differences in the relationship between loneliness and values. Specifically, we expect that the relationship between loneliness and SI values will differ between junior and senior high school students.

## 2 Methods

### 2.1 Participants and procedure

The sample for this study comprised two age groups: 6th graders from two junior high schools, and 10th graders from two senior high schools. All four schools involved in the study were located in Shanghai. Chinese middle and high school students were selected as participants for the study. This age group, 12–18 years of age, was chosen in consideration of other longitudinal and large-scale cross-sectional studies which have shown that loneliness among adolescents is of particular concern during these ages (Peng et al., 2023; Twenge et al., 2021). Adolescents are undergoing significant changes in their physical, psychological, and social roles, facing the multiple challenges of identity exploration, separation from their families of origin, and the establishment of independent relationships, all of which can contribute to increased loneliness. Additionally, as Chinese society is currently undergoing rapid change, increased academic and occupational competition, inadequate social skills, self-perception issues, and uncertainty about one’s future during middle and high school years can exacerbate adolescents’ experience of loneliness.

Study participants were pre-junior high school (6th grade) and senior high school (10th grade) students at T1. The reasons these age groups were chosen for this study were twofold. First, at these ages, students are all just entering middle or high school, and the changes in their roles and environments will impact their psychosocial adjustment, which includes both their level of loneliness and value identification. As such, the choice of using students of these ages is ideal for examining has both the practical and educational impacts of loneliness on adolescent development. Second, as a practical consideration for this longitudinal study, if other grades had been chosen, such as 8th and 11th grades, the T2 administration would have been conducted during the students’ third year of either junior or senior high school, at which time the students would be preparing for their Chinese high school or college entrance examinations, when academic pressure is greater, making it difficult for students to find the time to participate in the study.

Informed consent was obtained from the school leaders, the teachers, students’ parents, and the students involved in the experiment before the study commenced. Data collection was gathered using measurements which were administered to the class as a whole during their study period, with each session lasting approximately 30 min. During the data collection session, the researcher first explained the study’s title, objective, and methodology to the participants. Then, the researcher informed the participants that their involvement and completion of the questionnaires were voluntary and anonymous, and that their questionnaire responses would be anonymized and used solely for this study.

The first data collection point (T1) took place at the end of September 2022, and included 426 participants. Of this total, 239 were junior high school students, of which 49.10% were male, and the average age of this group was 12.93 years. The remaining 187 participants in the study were senior high school students, of which 43.08% were male, and with an average age of 16.85 years in this group. The second data collection point (T2) took place at the beginning of the following school year, in September 2023, at which point the participants had all been promoted to the next school grade. T2 included 403 participants, of which 224 were now in 7th grade (49.60% male), and 224 were now in 11th grade (42.50% male). The attrition rate was 5.40%, with the main reasons being absence from school due to illness (seven students), other activities scheduled at the time of data collection (nine students), and students having returned to their home schools (i.e., their parents had come to Shanghai from economically disadvantaged areas for work, but had now returned; seven students). ANOVA results showed no differences between the students who did or did not participate at T2 on six variables, *F*(12, 840) = 1.05, Wilks’ *λ* = 0.99, *p* = 0.35, η^2^ = 0.01.

### 2.2 Measures

#### 2.2.1 Values questionnaire

The values questionnaire used in this study was adopted from the Chinese Adolescents’ Values Questionnaire developed by Wang et al. (2018), which uses a total of 46 items to measure eight dimensions: social equality, collective responsibility (CR), rule abiding, family well-being, friendship, self-improvement (SI), fashion, and personal happiness. The two dimensions selected for this study we CR (e.g., “Everyone should consider the collective, and that the collective good is important”; seven items) and SI (e.g., “Everyone should always be trying to improve their abilities and skills”; six items). Items were presented as indirect measures, in that each item presented a description of a person, and respondents were asked to rate how similar they felt compared to the described person. Each items was rated using a five-point scale ranging from 1 (“Not at all like me”) to 5 (“Very much like me”). The Cronbach’s αs for CR and SI were 0.86 and 0.82 at T1, and 0.84 and 0.81 at T2, respectively.

#### 2.2.2 Loneliness scale

The Illinois Loneliness Questionnaire was used in this study to measure participants’ level of loneliness (Liu et al., 2018). The scale consists of 16 items (e.g., “I have nobody to talk to”), each of which is rated using a five-point scale ranging from 1 (“Not true at all”) to 5 (“Always true”). The mean score of all 16 items is calculated after converting the reverse-scored items, and a higher total score indicates a greater sense of loneliness. The Cronbach’s αs for the scale at T1 and T2 were 0.81 and 0.84, respectively.

### 2.3 Statistical analysis

First, descriptive statistics and bivariate correlations were calculated for all study variables. One-way ANOVA was conducted to examine gender and grade differences in key variables, with effect sizes reported as partial eta squared.

Second, longitudinal measurement invariance was tested for each construct across T1 and T2 using confirmatory factor analysis (CFA) with robust maximum likelihood estimation. Configural, metric, and scalar invariance were evaluated using chi-square difference tests and changes in CFI and RMSEA (ΔCFI ≤ 0.01 and ΔRMSEA ≤ 0.015 indicating invariance).

Third, cross-lagged panel models were estimated to examine the reciprocal relationships between loneliness and each value dimension (CR and SI) separately. Given that all scales demonstrated strong longitudinal invariance, latent variable cross-lagged models were specified with items parceled for the loneliness scale to improve model stability and parsimony. Three parcels were created for loneliness using the item-to-construct balance approach (Little et al., 2002), with items assigned to parcels based on factor loadings from an exploratory factor analysis. The CR and SI scales were modeled as latent variables with items as indicators.

Fourth, multiple-group cross-lagged models were conducted to test gender and grade differences. For each grouping variable, a baseline model with all paths freely estimated was compared to a constrained model with cross-lagged paths set equal across groups. Significant chi-square differences (*p* < 0.05) indicated group differences in the longitudinal relationships.

Model fit was evaluated using chi-square, CFI, TLI, and RMSEA. CFI and TLI values ≥ 0.90 and RMSEA values ≤ 0.08 were considered acceptable fit.

## 3 Results

### 3.1 Common method bias assessment

To examine potential common method bias, we conducted Harman’s single-factor test by entering all items from the three scales at both time points into an exploratory factor analysis. The unrotated solution revealed 12 factors with eigenvalues greater than 1, with the first factor explaining 14.3% of the variance, which is below the commonly used 40% threshold.

### 3.2 Descriptive statistics of variables

The missing data in this study were processed using Multiple Imputation.

The means and standard deviations of CR, SI, and loneliness are shown in Table 1 and correlation coefficients between variables are shown in Table 2. The six variables were all significantly correlated with one another. Among them, two values were significantly positively correlated with each other, and two values were significantly negatively correlated with loneliness.

**Table 1:** Descriptive statistics of variables *M(SD).*

Variables	7th		11th	
	Boy	Girl	Boy	Girl
T1CR	4.02 (0.88)	4.16 (0.88)	3.95 (0.81)	3.91 (0.65)
T2CR	4.03 (0.76)	4.12 (0.76)	3.96 (0.79)	3.90 (0.68)
T1SI	3.68 (0.86)	3.66 (0.90)	3.77 (0.72)	3.85 (0.73)
T2SI	3.83 (0.84)	3.91 (0.79)	3.97 (0.71)	4.00 (0.77)
T1 Loneliness	2.22 (0.65)	2.14 (0.62)	2.20 (0.39)	2.43 (0.47)
T2 Loneliness	2.25 (0.65)	2.16 (0.64)	2.30 (0.45)	2.47 (0.43)

CR, collective responsibility; SI, self-improvement.

**Table 2:** Correlation coefficients between variables.

Variables	1	2	3	4	5	6
1. T1CR	—					
2. T2CR	0.47^**^	—				
3. T1SI	0.52^**^	0.26^**^	—			
4. T2SI	0.28^**^	0.52^**^	0.51^**^	—		
5. T1 Loneliness	−0.46^**^	−0.38^**^	−0.32^**^	−0.28^**^	—	
6. T2 Loneliness	−0.37^**^	−0.50^**^	−0.26^**^	−0.35^**^	0.67^**^	—

All significant correlations in the table are at *p* < 0.01 level.

A repeated measures ANOVA (2 × 2 × 2) using CR as the dependent variable, measurement time (T1 and T2) as the within-subject variable, and gender and grade as the between-subject variables revealed a significant main effect of grade, *F*(1, 397) = 4.91, *p* = 0.02, *η^2^* = 0.01, with 7th grade students identifying more with CR than 11th grade students. Similarly, for SI, the repeated measures ANOVA showed only a significant main effect of time, *F*(1, 397) = 21.50, *p* < 0.01, *η^2^* = 0.05, indicating increased SI identification at T2. For loneliness, the repeated measures ANOVA revealed a significant main effect of time, *F*(1, 397) = 4.32, *p* = 0.04, *η^2^* = 0.01, with increased loneliness at T2 compared to T1, a significant main effect of grade, *F*(1, 397) = 9.30, *p* < 0.01, *η^2^* = 0.02, with higher loneliness in high school students compared to junior high students, and a significant gender × grade interaction, *F*(1, 397) = 7.61, *p* < 0.01, *η^2^* = 0.02. Specifically, there was no significant difference between 7th and 11th grade boys, SD = −0.02 (0.08), *p* = 0.84, whereas 11th grade girls reported significantly higher loneliness than 7th grade girls, SD = −0.30 (0.07), *p* < 0.01.

### 3.3 Cross-lagged models and age and gender differences

#### 3.3.1 Longitudinal measurement equivalence test

The results of model fitting for morphological equivalence, weak equivalence, and strong equivalence of each study variable are shown in Table 3. The results showed that the fitting indices of each model met the measurement requirements. According to the S-B chi-square test of variance and the change values of the fitted indices (ΔCFI, ΔRMSEA), loneliness, CR, and SI were shown to be strongly equivalent. These results suggested that the longitudinal relationships between the variables could be tested.

**Table 3:** Longitudinal equivalence test for each scale at T1–T2.

Model	*χ*^2^ (*df*)	CFA	TLI	RMSEA	Model comparisons	ΔCFI	ΔRMSEA
Loneliness							
M1: morphological equivalent	6.309 (5)	0.998	0.996	0.023			
M2: weak equivalence	8.148 (7)	0.999	0.998	0.017	M2–M1	0.000	−0.004
M3: strict equivalence	11.101 (9)	0.999	0.998	0.021	M3–M2	0.000	0.004
CR							
M1: morphological equivalent	157.882 (69)	0.949	0.921	0.049			
M2: weak equivalence	163.798 (75)	0.949	0.939	0.049	M2–M1	0.000	−0.002
M3: strict equivalence	172.337 (81)	0.950	0.944	0.047	M3–M2	−0.001	−0.002
SI							
M1: morphological equivalent	108.577 (47)	0.916	0.912	0.046			
M2: weak equivalence	125.247 (52)	0.918	0.916	0.048	M2–M1	0.000	−0.005
M3: strict equivalence	138.486 (57)	0.908	0.907	0.049	M3–M2	−0.009	−0.003

#### 3.3.2 Cross-lagged models

First, two cross-lagged models were constructed, one for loneliness and CR, and one for loneliness and SI. The models fit well: loneliness and CR, *χ*^2^ = 506.77, *df* = 164, χ^2^/*df* = 3.09, RMSEA = 0.07, CFI = 0.91, TLI = 0.90; and loneliness and SI, *χ*^2^ = 332.21, *df* = 127, *χ*^2^*/df* = 2.62, RMSEA = 0.06, CFI = 0.93, TLI = 0.92.

**Figure 1:**
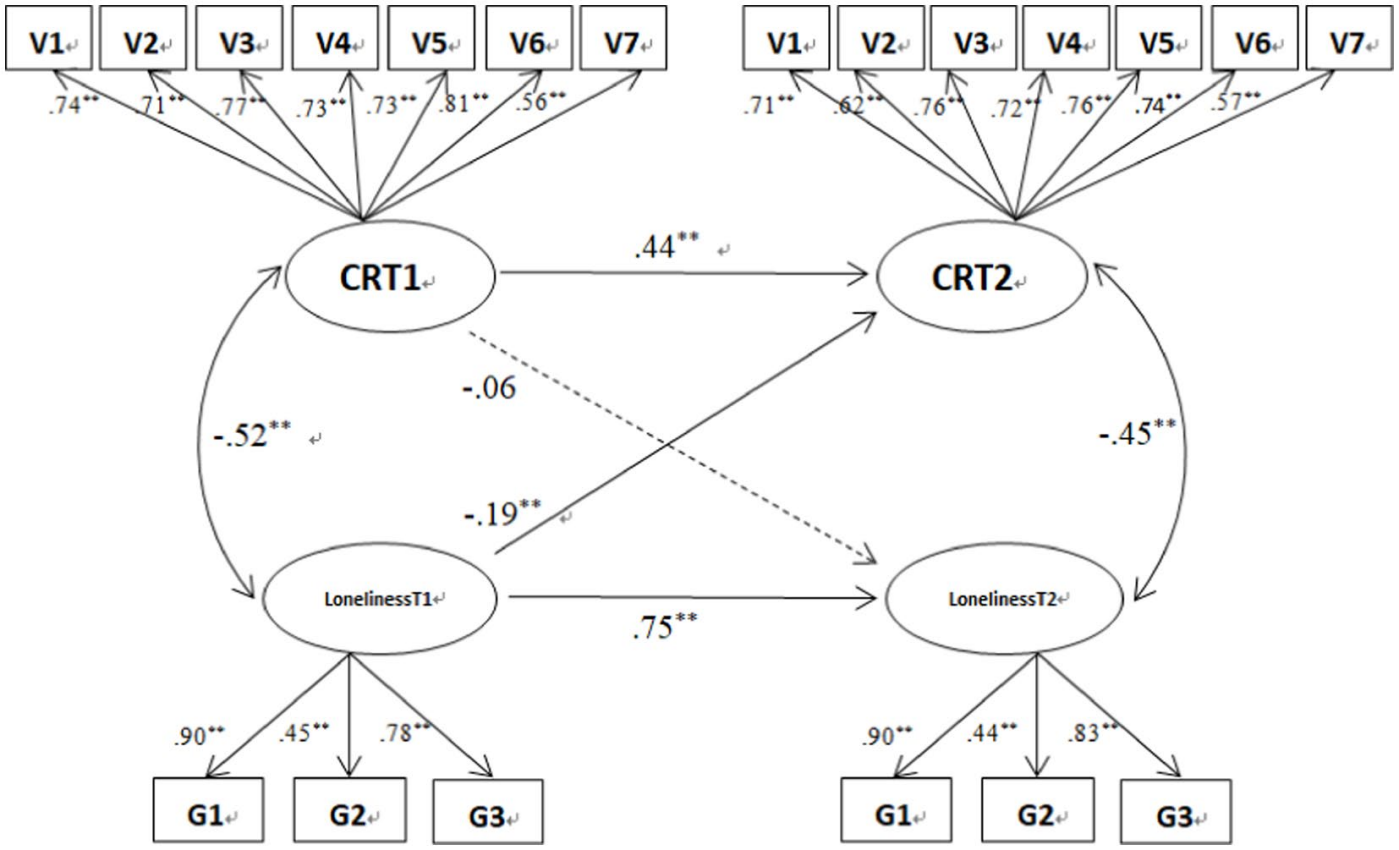
Cross-lagged model of loneliness and CR (numbers in the figure are normalized path coefficients). **p < 0.01.

**Figure 2:**
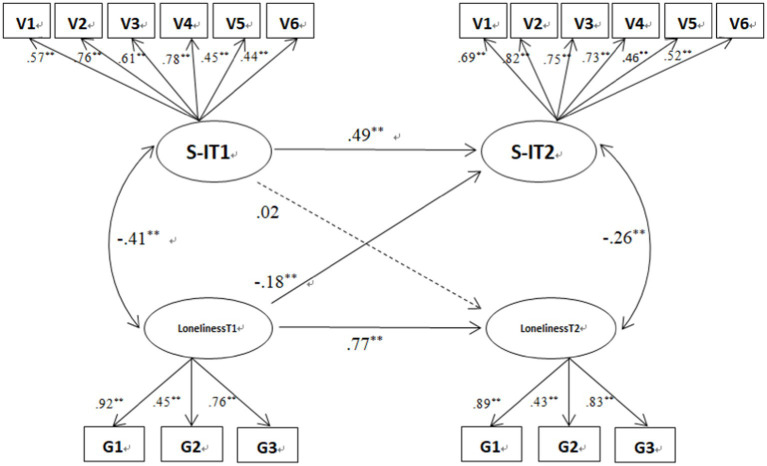
Cross-lagged model of loneliness and SI. **p < 0.01.

Cross-lagged paths of the two models showed that loneliness at T1 significantly and negatively predicted CR at T2, *β* = −0.19, *SE* = 0.06, *p* < 0.01. Loneliness at T1 significantly and negatively predicted SI at T2, *β* = −0.18, *SE* = 0.06, *p* < 0.01. Cross-lagged and autoregressive paths of the models are shown in Figures 1, 2.

#### 3.3.3 Gender and age differences in the cross-lagged models

Grade and gender differences were then examined using the two models (i.e., loneliness–CR and loneliness–SI) using grade and gender as grouping variables for the multiple cross-lagged models. Initially, age differences in the relationship between loneliness and the values were examined by constructing restricted models that set the cross-lagged paths to be equivalent, while the autoregressive coefficients of the variables were estimated freely, then comparing the fit of the restricted model to the fit of the initial model in which all-paths were estimated freely. Because the focus of this study was on the effects of loneliness on CR and SI, grade and gender differences in the autoregressive coefficients of the three variables were not compared. The coefficients were set so that the T1–T2 autoregressive coefficients of the three variables were now set as free estimates in both restricted and free estimated models. The same procedure was followed in all of the subsequent operations.

The difference between the restricted and freely estimated model indicators for loneliness–CR were Δ*χ*^2^ = 1.82, Δ*df* = 2.0, and *p* = 0.40, indicating no significant difference due to age in the interaction between loneliness and CR.

For loneliness–SI, the difference between the restricted and freely-estimated model indicators was Δ*χ*^2^ = 7. 91, Δ*df* = 2.0, *p* = 0.02, suggesting that there was a significant difference in the loneliness–SI interaction according to grade. Specifically, the 7th graders’ identification with SI at T1 negatively predicted their loneliness at T2, whereas the 11th graders’ identification with SI at T1 significantly positively predicted their loneliness at T2. The differences between SI_T1_–loneliness_T2_ of the 7th and 11th grade students was −0.25, *SE* = 0.10, *p* = 0.01. The path coefficient for the 7th graders was −0.11, *SE* = 0.07, *p* = 0.09, while the path coefficient for the 11^th^ graders was 0.17, *SE* = 0.08, *p* = 0.04. There were no differences due to age in loneliness at T1 with regards to participants’ identification with SI at T2, 1 year later: loneliness_T1_–SI_T2_ (grades 7 to 11) = −0.11, *SE* = 0.11, *p* = 0.34.

Gender differences in the relationship between loneliness and the two values were also evaluated. The difference between the restricted and unconstrained estimation model indicators of loneliness–CR was Δχ^2^ = 0.84, Δ*df* = 2.0, *p* = 0.66, indicating no significant gender differences in the loneliness–CR interaction.

The difference between the restricted and unconstrained model indicators for loneliness–SI was Δχ^2^ = 6.03, Δ*df* = 2.0, *p* = 0.049, indicating a significant gender difference in the loneliness–SI interaction. Loneliness at T1 significantly and negatively predicted identification with SI at T2 among the male students, but not among the female students. Loneliness_T1_–SI_T2_ (males–females) = −0.26, *SE* = 0.11, *p* = 0.02. The path coefficient for the male students was −0.30, *SE* = 0.08, *p* < 0.001, and the path coefficient for the female students was 0.02, *SE* = 0.08, *p* = 0.79. No gender difference was found between identification with SI at T1 and loneliness at T2, 1 year later: SI_T1_–loneliness_T2_ (males–females) = 0.01, *SE* = 0.11, *p* = 0.96.

## 4 Discussion

### 4.1 The impact of loneliness on value identity

The results of this study found that loneliness at T1 negatively predicted CR and SI values at T2, meaning that the more lonely adolescents felt at the first measurement point, the less likely they were to identify with either value at the second measurement point 1 year later. When adolescents experience loneliness over a long period of time, they develop negative emotions regarding interpersonal relationships, such as feelings of rejection or non-acceptance, as well as withdrawal and aggressive behaviors (Wang et al., 2026; Xiong et al., 2021). As a result, adolescents may become alienated from the group, as they are unwilling to interact with others and participate in group life. This may lead to a lower identification with the value of CR, as adolescents may also experience emotions such as disappointment, despair, decadence, or emptiness, and become addicted to the Internet or their cell phone as a means of distraction (Li et al., 2025; Zhang et al., 2019). They may act depressed or demoralized, losing motivation to achieve, curiosity, a competitive spirit, or assertiveness. Some young people might even begin to pursue “Sang” or “Emo” subcultures, while even others may become “Buddhist youth” or “lying youth,” all of which might lead to a decrease in identification with the value of SI (Lan and Wang, 2025).

In the present study, the effect of loneliness on CR and SI was more consistent with the path of the values’ unconscious change over time. According to Bardi and Goodwin (2011), values can change through two types of pathways: automatic or intentional. As emotions can often have diffuse and subconscious influences on human perceptions and behaviors, the values one identifies with can be changed through methods such as priming, consistency-maintenance, direct persuasion, adaptation, and group identification. Priming and consistency-maintenance, specifically, can provide some explanation as to the effect of loneliness on values. In general, individuals strive to have alignment between their attitudes, behaviors, and self-concept; and aligning one’s values with their self-concept is particularly common as one’s values are central to their self-concept (Bardi and Goodwin, 2011). When one perceives consistent environmental cues such as being alone or in an excluded group setting, this can lead them to change their values, as the individual will no longer identify with group-self oriented values in order to conform to their unaccepted or incompetent self-concept.

### 4.2 Age and gender differences in the relationship between loneliness and values

Our results also indicated that 7th grade students’ identification with SI at T1 negatively predicted their loneliness at T2. This could be due to the fact that identification with SI was positively associated with academic achievement (Wang et al., 2018; Li X. R. et al., 2018; Li D. et al., 2018), and that middle school students’ academic performance and ability to improve are particularly noticed and recognized by their parents or teachers at this age, which may somehow reduce or alleviate their loneliness. For example, academic performance has been shown to negatively predict adolescent loneliness through teacher acceptance (Liu et al., 2018), and parental encouragement of adolescent autonomy in choices and decision-making has been associated with reduced adolescent loneliness (Li et al., 2019). For students in 11th grade, unlike for those in 7th grade, identification with SI at T1 positively predicted their loneliness 1 year later at T2. This could be because high school students face greater academic pressure and competition, which can reduce their interactions with peers and lead to an increase in loneliness. Indeed, some studies have found that peer competition and a potentially comparative climate can lead to a variety of school adjustment problems, including anxiety, decreased well-being, and peer conflict. In other words, identification with SI may intensify peer competition and comparison, making it more difficult to sense friendship and care from peers, thereby increasing loneliness. Furthermore, high school students are more motivated by autonomy, becoming increasingly interested in having independence from their families. High school students are more aware than middle school students that studying diligently is primarily for their own development, and not for external recognition. Consequently, parental and teacher attention or approval do not fully reduce or alleviate feelings of isolation they may experience due to the fact that their time is preoccupied by studying.

These divergent patterns may also reflect different stages of identity formation. As identity development involves ongoing processes of exploration and commitment (Maehler and Hernández-Torrano, 2025), the social context and sources of validation differ between junior and senior high students. For junior high students, identity exploration is more social-oriented, with peers and adults providing external validation and guidance (Rageliene, 2016). In contrast, for senior high students, identity formation becomes more autonomous and internally driven, characterized by greater agency and self-determination (Schwartz et al., 2005). The pursuit of SI during this developmental stage may represent individual achievement rather than social connection, which may explain why SI values are associated with increased loneliness during this transition.

The results of this study showed that adolescents were more likely to identify with SI at T2 than they did at T1. This is consistent with the findings of previous studies which have demonstrated that adolescent identification with values such as achievement and self-direction increases with age. One study concluded that during adolescence, individuals will identify increasingly with self-oriented values and decreasingly with other-oriented values (Daniel et al., 2020). These results could also be related to certain characteristics of the Chinese values identity, as some domestic studies have indicated that compared to Western values, Chinese values are more holistic and integrated and that although they emphasize enterprise and self-discipline, Chinese values are always “people-centered” (Wang et al., 2018; Li X. R. et al., 2018; Li D. et al., 2018), with a fundamental goal of the harmonious development of the group and society. Therefore, the relationship between SI and loneliness may essentially reflect the relationship between the self and society in the development of adolescent values. In other words, the three types of values—individual striving, collectivism, and relationality—present a state of integration and balance (Yang and Huang, 2025). When their values are conducive to harmony between the self and society, their values promote their psychosocial adaptation; when their values deviate from harmony between the self and society, their values undermine their psychosocial adaptation.

In this study, gender differences were seen in the effect of loneliness on SI, with the loneliness of the male students at T1 significantly and negatively predicting their SI values at T2, 1 year later. Cultural expectations of males generally emphasize their abilities to solve problem on their own and maintain emotional control, however when they experience psychological problems or stressors, these expectations can then lead to boys being less well-adjusted. Boys have also been found to avoid problems by immersing themselves in online gaming and Internet use (Zhang et al., 2023). Boys experience certain developmental disadvantages during adolescence compared to girls, including lower levels of self-control and self-esteem (Zhang et al., 2023). Furthermore, Chinese cultural values expect boys to be strong, independent, and confident, and in terms of parenting style, parental supervision and warmth directed toward boys are substantially lower than they are toward girls. As a result, boys bear higher social expectations while failing to receive care, encouragement, or advice from their parents. This long-term experience of emotional or psychological isolation can result in males becoming more likely to lack goals or motivation, thus becoming less likely to identify with SI.

In addition to the longitudinal pathways examined in this study, alternative explanations and potential mechanisms should be considered. First, while our primary focus was on loneliness as an antecedent of value development, the cross-lagged design also revealed bidirectional effects, particularly the finding that SI at T1 predicted loneliness at T2 in opposite directions for junior versus senior high students. This suggests that value identification may also influence loneliness through different mechanisms depending on developmental stage.

Second, several third variables may contribute to the observed relationships. For example, depression may mediate the association between loneliness and values, given documented links between loneliness, depressive symptoms, and value-related behaviors (Chen F. et al., 2023; Chen Z. et al., 2023). Parenting practices, including autonomy support and warmth, may influence both loneliness and value socialization, as parental relationships shape adolescents’ sense of connection and their internalization of societal values (Li et al., 2019). Peer relationships and peer acceptance may similarly affect both loneliness trajectories and values, as peer groups provide contexts for value enactment and social belonging.

Third, potential mediators and moderators warrant further investigation. Coping style may mediate the relationship between loneliness and values; adolescents experiencing loneliness may adopt passive or avoidant coping strategies, which could reinforce both loneliness and de-identification from pro-social values. School climate, including classroom competitiveness and teacher support for collaboration, may moderate the effects of loneliness on values by providing contexts that either exacerbate or buffer the impact of social isolation on value development.

## 5 Limitations and implications

Despite its contributions to the literature, this study does have certain limitations. First, the measurement interval was relatively small, as was the sample selection in this study. Furthermore, they should select a larger and more diverse sample. Second, there may be additional mediating or moderating variables in the relationship between loneliness and CR and SI values, such as school climate, autonomy, or coping style. The findings of the study also have practical implications for addressing adolescent mental health, values education, and the development of interventions.

## Data Availability

The datasets presented in this article are not readily available because the dataset forms part of a doctoral dissertation. It constitutes a project of the research group and remains the property of the group. Use of the dataset cannot be disclosed and requires confidentiality. Requests to access the datasets should be directed to Xiaofeng Wang, lndafeng@163.com.
